# Supplementary material for: PhaseStain: the digital staining of label-free quantitative phase microscopy images using deep learning
Source: Light Sci Appl. 2019 Feb 6;8:23. doi: 10.1038/s41377-019-0129-y (PMC6363787; doi:10.1038/s41377-019-0129-y)
Supplement: Supplementary file 1 — Supplementary Information [file 41377_2019_129_MOESM1_ESM.pdf]

Supplementary Information

PhaseStain: the digital staining of label-free quantitative phase microscopy images using deep learning

Yair Rivenson[1,2,3†], Tairan Liu[1,2,3†], Zhensong Wei[1,2,3†], Yibo Zhang[1,2,3], Kevin de Haan[1,2,3], Aydogan Ozcan[1,2,3,4]*

[1]*Electrical Engineering Department, University of California, Los Angeles, CA, 90095, USA.*

[2]*Bioengineering Department, University of California, Los Angeles, CA, 90095, USA.*

[3]*California NanoSystems Institute (CNSI), University of California, Los Angeles, CA, 90095, USA.*

[4]*Department of Surgery, David Geffen School of Medicine, University of California, Los Angeles, CA, 90095, USA.*

*Correspondence: ozcan@ucla.edu

(a) *Generator*

Down path

$$QPI\ input(P\times P)\xrightarrow{\text{LReLU}(Conv())}(P\times P\times 32)\xrightarrow{\text{LReLU}(Conv())}(P\times P\times 32)\xrightarrow{\text{LReLU}(Conv())}(P\times P\times 64)\xrightarrow{Add\ padded\ input(P\times P\times 64)}s1(P\times P\times 64)\xrightarrow{Max\ Pooling}$$

$$s2(\frac{P}{2}\times\frac{P}{2}\times 64)\xrightarrow{\text{LReLU}(Conv())}(\frac{P}{2}\times\frac{P}{2}\times 96)\xrightarrow{\text{LReLU}(Conv())}(\frac{P}{2}\times\frac{P}{2}\times 96)\xrightarrow{\text{LReLU}(Conv())}(\frac{P}{2}\times\frac{P}{2}\times 128)\xrightarrow{Add\ padded\ s2(\frac{P}{2}\times\frac{P}{2}\times 128)}s3(\frac{P}{2}\times\frac{P}{2}\times 128)\xrightarrow{Max\ Pooling}$$

$$s4(\frac{P}{4}\times\frac{P}{4}\times 128)\xrightarrow{\text{LReLU}(Conv())}(\frac{P}{4}\times\frac{P}{4}\times 192)\xrightarrow{\text{LReLU}(Conv())}(\frac{P}{4}\times\frac{P}{4}\times 192)\xrightarrow{\text{LReLU}(Conv())}(\frac{P}{4}\times\frac{P}{4}\times 256)\xrightarrow{Add\ padded\ s4(\frac{P}{4}\times\frac{P}{4}\times 256)}s5(\frac{P}{4}\times\frac{P}{4}\times 256)\xrightarrow{Max\ Pooling}$$

$$s6(\frac{P}{8}\times\frac{P}{8}\times 256)\xrightarrow{\text{LReLU}(Conv())}(\frac{P}{8}\times\frac{P}{8}\times 384)\xrightarrow{\text{LReLU}(Conv())}(\frac{P}{8}\times\frac{P}{8}\times 384)\xrightarrow{\text{LReLU}(Conv())}(\frac{P}{8}\times\frac{P}{8}\times 512)\xrightarrow{Add\ padded\ s6(\frac{P}{8}\times\frac{P}{8}\times 512)}s7(\frac{P}{8}\times\frac{P}{8}\times 512)\xrightarrow{Max\ Pooling}$$

$$Down\ Path\ Output(\frac{P}{16}\times\frac{P}{16}\times 512)$$

Connection layer

$$Down\ Path\ Output(\frac{P}{16}\times\frac{P}{16}\times 512)\xrightarrow{\text{LRelu}(Conv())}Up\ Path\ Input(\frac{P}{16}\times\frac{P}{16}\times 512)$$

Up path

$$Up\ Path\ Input(\frac{P}{16}\times\frac{P}{16}\times 512)\xrightarrow{Bilinear\ Up\ Sample\ and\ Concatenate\ s7}(\frac{P}{8}\times\frac{P}{8}\times 1024)$$

$$\xrightarrow{\text{LReLU}(Conv())}(\frac{P}{8}\times\frac{P}{8}\times 640)\xrightarrow{\text{LReLU}(Conv())}(\frac{P}{8}\times\frac{P}{8}\times 640)\xrightarrow{\text{LReLU}(Conv())}(\frac{P}{8}\times\frac{P}{8}\times 256)\xrightarrow{Bilinear\ Up\ Sample\ and\ Concatenate\ s5}(\frac{P}{4}\times\frac{P}{4}\times 512)$$

$$\xrightarrow{\text{LReLU}(Conv())}(\frac{P}{4}\times\frac{P}{4}\times 320)\xrightarrow{\text{LReLU}(Conv())}(\frac{P}{4}\times\frac{P}{4}\times 320)\xrightarrow{\text{LReLU}(Conv())}(\frac{P}{4}\times\frac{P}{4}\times 128)\xrightarrow{Bilinear\ Up\ Sample\ and\ Concatenate\ s3}(\frac{P}{2}\times\frac{P}{2}\times 256)$$

$$\xrightarrow{\text{LReLU}(Conv())}(\frac{P}{2}\times\frac{P}{2}\times 160)\xrightarrow{\text{LReLU}(Conv())}(\frac{P}{2}\times\frac{P}{2}\times 160)\xrightarrow{\text{LReLU}(Conv())}(\frac{P}{2}\times\frac{P}{2}\times 64)\xrightarrow{Bilinear\ Up\ Sample\ and\ Concatenate\ s1}(P\times P\times 128)$$

$$\xrightarrow{\text{LReLU}(Conv())}(P\times P\times 80)\xrightarrow{\text{LReLU}(Conv())}(P\times P\times 80)\xrightarrow{\text{LReLU}(Conv())}(P\times P\times 32)\xrightarrow{Conv()}Output(P\times P\times 3)$$

(b) *Discriminator*

$$GO/GT(P\times P\times 3)\xrightarrow{\text{LRelu}(Conv())}(P\times P\times 64)$$

$$\xrightarrow{\text{LReLU}(Conv())}(P\times P\times 64)\xrightarrow{\text{LReLU}(ConvWstride2())}(\frac{P}{2}\times\frac{P}{2}\times 128)$$

$$\xrightarrow{\text{LReLU}(Conv())}(\frac{P}{2}\times\frac{P}{2}\times 128)\xrightarrow{\text{LReLU}(ConvWstride2())}(\frac{P}{4}\times\frac{P}{4}\times 256)$$

$$\xrightarrow{\text{LReLU}(Conv())}(\frac{P}{4}\times\frac{P}{4}\times 256)\xrightarrow{\text{LReLU}(ConvWstride2())}(\frac{P}{8}\times\frac{P}{8}\times 512)$$

$$\xrightarrow{\text{LReLU}(Conv())}(\frac{P}{8}\times\frac{P}{8}\times 512)\xrightarrow{\text{LReLU}(ConvWstride2())}(\frac{P}{16}\times\frac{P}{16}\times 1024)$$

$$\xrightarrow{\text{LReLU}(Conv())}(\frac{P}{16}\times\frac{P}{16}\times 1024)\xrightarrow{\text{LReLU}(ConvWstride2())}(\frac{P}{32}\times\frac{P}{32}\times 2048)$$

$$\xrightarrow{Average\ Pooling}(2048\ vector)\xrightarrow{\text{LReLU}(FC())}(2048\ vector)$$

$$\xrightarrow{Sigmoid(FC())}(scalar):real\ /\ fake$$

Supplementary Table 1. Detailed GAN architecture. LReLU: Leaky ReLU, Conv: convolutional layer with a stride of 1, FC: fully-connected layer, ConvWstride2: convolutional layer with stride 2, GO: generated output, GT: ground truth image.
